# Case report: ^125^I seed implantation for rare malignant solitary fibrous tumor in the pelvic cavity: a case report

**DOI:** 10.3389/fonc.2022.884491

**Published:** 2022-08-01

**Authors:** Zhen Gao, Huimin Yu, Xuemin Di, Jinxin Zhao, Yansong Liang, Zezhou Liu, Juan Wang, Hongtao Zhang

**Affiliations:** Department of Oncology, The Hebei General Hospital, Shijiazhuang, China

**Keywords:** ^125^I seeds, brachytherapy, malignant solitary fibrous tumor, recurrence, MSFT

## Abstract

Solitary fibrous tumor (SFT) is a rare spindle cell tumor, benign or low-grade malignant, with an extremely low possibility of occurrence of malignant solitary fibrous tumor (MSFT). Surgery is an effective way for treating SFT, but it is often difficult to resect completely due to a large size, with a high recurrence rate and mortality rate after operation. Additionally, SFT is relatively resistant to chemotherapy, and there is a lack of effective systemic drug treatment. These lead to certain difficulties in the treatment of SFT. We report a case of a rare MSFT in the pelvic cavity. With a history of recurrence after two surgeries, this patient underwent surgical removal combined with ^125^I seed implantation at our hospital in the context that the tumor could not be completely removed because it was large and adhered to surrounding tissues; after up to 43 months of progression-free survival (PFS), the patient underwent ^125^I seed implantation alone, and achieved a complete remission, with a PFS up to 35 months. ^125^I seed implantation can be a safe and effective treatment option for unresectable MSFT as well as a potential solution to repeated local recurrence.

## Introduction

Solitary fibrous tumor (SFT) is a rare spindle cell tumor originating from CD34-positive dendritic mesenchymal cells (1). It has three typical primary sites: the pleura, the meninges, and extrathoracic soft tissues (2), and can also occur at a wide variety of sites including the head and neck, extremities, kidneys, liver, prostate, adrenal glands, and skin and soft tissues (3–5). It usually affects middle-aged people (6). Generally, SFT, benign or low-grade malignant, behaves in a benign or borderline fashion, and in very rare cases, it can develop as a malignant tumor (7). For the treatment of SFT, surgery is an effective approach, but a complete resection often becomes difficult due to a large size of tumor, and postoperative relapse and death events become frequent; SFT is relatively resistant to chemotherapy, and thus there is a lack of effective systemic drugs, making the treatment further challenging. While previous literature has mostly focused on the imaging and molecular pathological features of SFT (8, 9), there are few reports on the adjuvant therapy for MSFT, and there is no treatment guideline for MSFT. ^125^I seeds, a radioactive material of brachytherapy, can be placed into tumors during surgery or under imaging guidance, and then continuously release low-energy gamma rays to destroy the DNA double strands of tumor cells. This approach can increase the radiation dose to the tumor target, and dramatically decrease the dose to surrounding normal tissues, showing great advantages of safety, minimal invasion, definite benefit, few complications, low level of radioactive contamination, and convenience for protection. In recent years, it has been used for treating solid tumors at various sites and has achieved good performance (10–13). We report a case of MSFT undergoing ^125^I seed implantation and attaining a good therapeutic effect, and we have yet to find such reports in China and abroad.

## Case report

**Figure 1:**
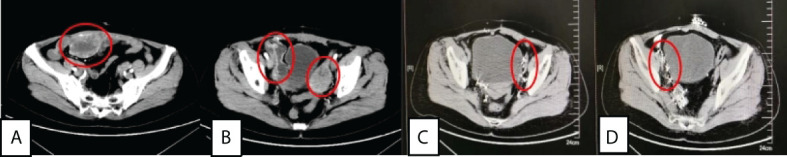
**(A)** and **(B)** were abdominal wall tumor(7×5×8 cm), left pelvic tumor(5.5×3.5×7 cm) and right iliac paravascular tumor(8.5×3×5 cm) before treatment, while **(C)** and **(D)** were left pelvic and right iliac paravascular tumors without recurrence 43 months after surgery+^125^I seed implantation.

**Figure 2:**
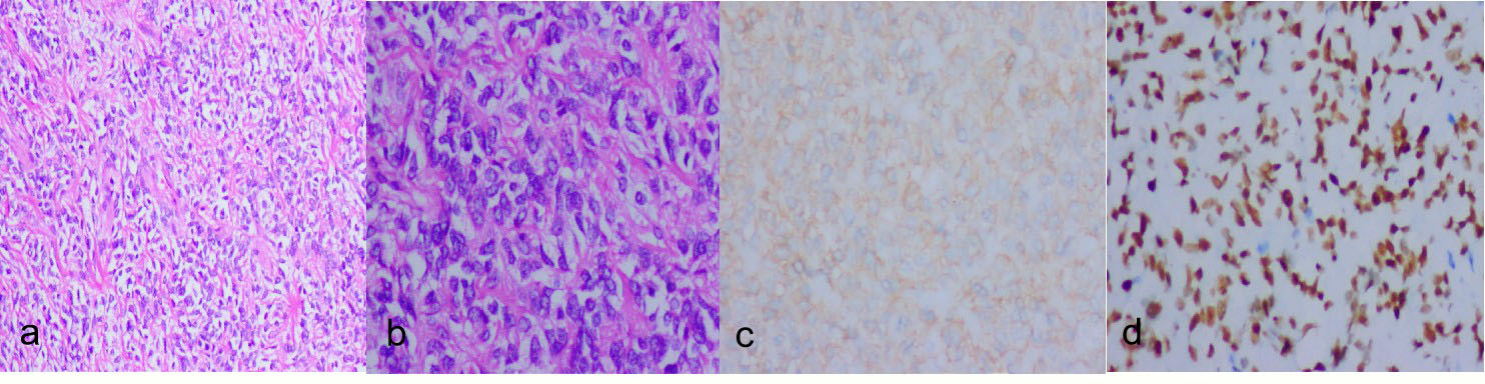
**(A)**:hypercellular areas(HE ×100). **(B)**:hypercellular area with atypical nuclei and some evident mitotic figures(HE ×200). **(C)**:Tumor cells are diffusely positive for CD99(IHC×200). **(D)**:Tumor cells are diffusely positive for STAT6 (IHC ×100).

**Figure 3:**
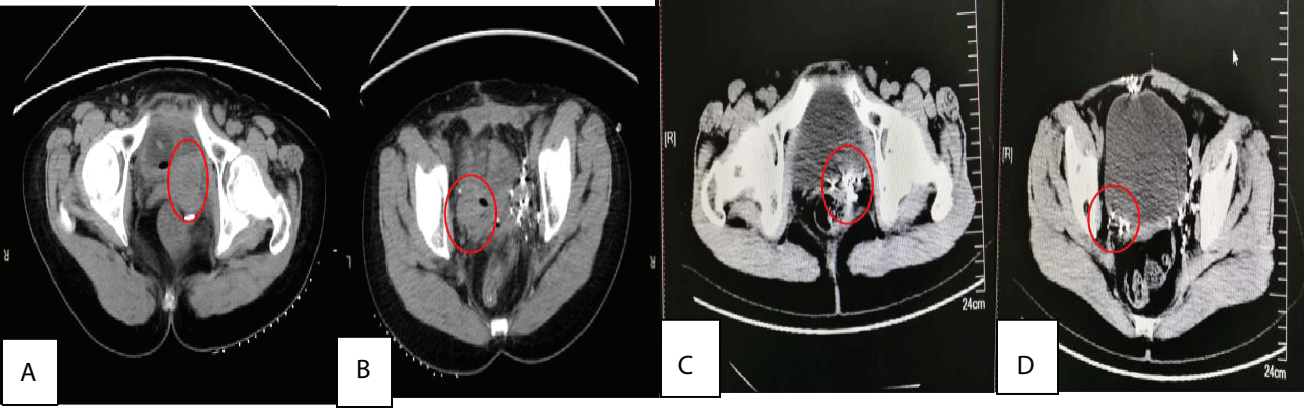
**(A)** and **(B)** were behind bladder tumors before treatment with size of about 4×3×5.0 cm, 3.0×2.0×2.5cm respectively, and **(C)** and **(D)** were two years after ^125^I seed implantation.

A 57-year-old women was first admitted to our hospital on June 29, 2015, complaining that she had undergone surgery for pelvic masses 7 years before admission and had experienced a recurrence 1 year before admission. In 2008, the patient initially presented with irregular vaginal bleeding; her gynecological ultrasound suggested multiple uterine fibroids, and her pelvic magnetic resonance imaging displayed abnormal signal intensity in the left wall of the cervix. In September 2008, she underwent total hysterectomy and removal of the tumor between the vagina and bladder; postoperative pathology demonstrated multiple uterine fibroids and vaginal fibroma. At the beginning of 2014, the patient felt distension in the lower abdomen, with multiple pelvic masses on computed tomography (CT). On April 8, 2014, she underwent pelvic mass resection; postoperative pathology showed a borderline or low-grade malignant spindle cell tumor, consistent with SFT, and the immunohistochemistry results were as follows: Ki-67 (5% positive), Bcl-2 (-), Desmin (-), SMA (-), DOG-1 (-), CD68 (-), CD117 (-), S-100 (-), CD34 (blood vessels +), CD99 (+), and Calretinin (-). Two months later, her CT displayed a walnut-size mass each under the abdominal wall and in the vaginal stump, with no specific treatment. On June 29, 2015, the patient visited our hospital and had abdominal and pelvic CT examination, which indicated multiple space-occupying lesions in the abdominal wall and pelvic cavity (**Figure 1**). On July 7, 2015, she underwent exploratory laparotomy under general anesthesia; the tumors were distributed in the right wall of the abdomen, in the left pelvic cavity, near the right iliac vessels, and behind the bladder; considering the large size of tumors as well as adhesion between the abdominal wall tumor and the small intestine and between the pelvic tumors and iliac vessels, the tumors could not be resected completely, so in addition to removal of the tumors in the abdominal wall and pelvic cavity. Postoperative microscopic observation: the tumor cells were spindle with unclear boundaries and diffuse hyperplasia, with large, fusiform, round or oval nuclei, pathological mitosis of 5 per 10HPF, intercellular collagen fibers, scattered lymphocytes, bleeding and necrosis, and small vascular hyperplasia. Postoperative pathology showed recurrent solitary fibrous tumor, considered low-grade malignancy, and the immunohistochemistry results were as follows:CD99 (Weak +), CD34 (blood vessels+), SMA (-), Bcl-2 (+), Vimentin(+), Wide CK(-), S-100(-), Desmin(-), CR(-), MC(-), Ki-67 (10% positive) (**Figures 2A–C**).^125^I seed implantation was performed, in which 30 seeds (0.3 mCi) were implanted into the tumor bed near the right iliac vessels, with D_90_ of 62.0 Gy, and 32 seeds (0.5 mCi) into the tumor bed of the left pelvic cavity, with D_90_ of 58.0 Gy; the operation was smooth, and postoperative pathology came back as a recurrent low-grade MSFT. In August 2015, a residual tumor was observed on CT in the posterior wall of the bladder. On August 27, 2015, under the guidance of CT, 50 ^125^I seeds (0.3 mCi) were placed percutaneously into the tumor in the left posterior wall of the bladder, with D_90_ of 64.8 Gy measured post implant. On September 21, 2015, under CT guidance, 28 ^125^I seeds (0.3 mCi) were implanted into the tumor in the right posterior wall of the bladder, with D_90_ of 49.7 Gy measured post implant. Dose-volume histogram parameters were applied for the evaluation of target volume and organs at risk (OARs).The patient’s regular examinations after implants revealed a complete remission (**Figure 3**). No major complication (fever, hemorrhage, bone marrow suppression, liver/kidney dysfunction, skin/mucosal radiation reaction, radiation enteritis, or cystitis) was observed during our follow-up period. No seeds migrated to other tissues or organs.

**Figure 4:**
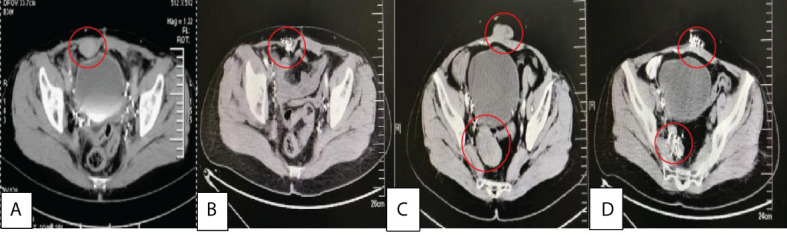
**(A)** was the recurrence of abdominal wall tumor with a size of about 4.0×2.5×4.5 cm before treatment in 2016/1, **(B)** was 6 months after ^125I^ seed implantation, **(C)** was the recurrence of abdominal wall tumor(3.6×1.5×3.0 cm) and behind bladder tumors(5.0×3.0×5.0 cm) before treatment in 2018/10, **(D)** was 4 months after ^125^I seed implantation.

In January 2016, CT showed that a tumor recurred in the abdominal wall, with a size of about 4.0×2.5×4.5 cm. On January 6, 2016, she underwent implantation of 29 ^125^I seeds (0.4 mCi) into the wall tumor, with D_90_ of 77.8 Gy. Her regular examinations thereafter demonstrated a complete remission all the way until October 2018, when CT displayed recurrent tumors in the abdominal wall and pelvic cavity. On October 16, 2018, with the help of CT guidance, 54 ^125^I seeds (0.5 mCi) were embedded into the SFT (5.0×3.0×5.0 cm) under the right side of the bladder, with D_90_ of 97.6 Gy; on October 23, 2018, 30 ^125^I seeds (0.5 mCi) were embedded into the SFT (3.6×1.5×3.0 cm) in the abdominal wall, with D_90_ of 101.3 Gy. The patient achieved a complete remission, no complications were found, and the brachytherapy was well tolerated by the patient, The patient had no recurrence of abdominal and pelvic tumors during the 4-month follow-up (**Figure 4**).

## Discussion

The diagnosis of SFT mainly depends on the pathologic features and immunophenotypes. Histologically the tumor was composed of abundant and dense cells, which were separated by rope like collagenous fibrous stroma. The tumor cells were spindle to short-spindle shaped with marked nuclear atypia and increased mitotic activity. The tumor cells arranged in fascicular or swirl pattern. In focal areas, hemangiopericytoma-like structure, coagulative necrosis, focal hemorrhage and invasive margin were presented. MSFT is usually grossly indistinguishable from conventional forms, but it may also show a more irregular cut surface, with evidences of necrotic areas or infiltration of the nearest tissues (14). Immunohistochemistry plays a pivotal role in differentiating SFTs from other spindle cell mesenchymal tumors. SFTs are immunoreactive to Vimentin, CD34, CD99 and bcl-2 and they are negative for actin, desmin (in smooth muscle tumors), keratin and CD117 (in GISTs) (15, 16). Recently, the discovery of the NAB2-STAT6 fusion gene in SFT led to development of a STAT6 antibody that is a reliable immunohistochemical marker with a high level of sensitivity and specificity. Therefore, nuclear expression of STAT6 is currently the most useful marker (17).In this case, we do not perform STAT6 immunohistochemistry initially, because the marker was not widely used at that time. But some years later, we perform STAT6 immunohistochemistry and the diffuse STAT6 nuclear positivity further confirmed the diagnosis (**Figure 2D**). SFT is mostly benign. Malignant transformation may also occur within histologically benign SFTs even after several years of diagnosis (18). The diagnosis of malignant solitary fibrous tumors in the 2020 edition of WHO soft tissue pathology classification continues to follow the previous criteria (19), mainly including: (1) The presence of high cellularity;(2) Cellular pleomorphism; (3) high mitotic count, usually more than 4/10HPF; (4) neoplastic necrosis. The case met the diagnostic criteria of MSFT.

Generally, the most effective treatment for SFT is surgical resection. However, in most cases, when patients feel discomfort and visit doctors, tumors have grown to large masses — beginning to press on adjacent organs to produce the warning symptoms — and there are usually abundant blood vessels and collateral circulation around the tumors, often making surgery difficult. Relevant literature has shown that patients with benign SFT undergoing surgical removal have a median 10-year overall survival rate of 54%-89% (20–23), and 20%-30% of the patients will experience local recurrence or metastasis. Reoperation can be considered in some patients with advanced SFT, but the recurrence rate and mortality rate are still very high. SFT is generally insensitive to chemotherapy, and sensitive to only a few chemotherapy drugs, according to European and American studies. Therefore, it is important to find a safe and effective treatment approach based on the characteristics of SFT. We report our application of ^125^I seed implantation in MSFT, providing a novel solution to the treatment of the disease.

This patient was initially diagnosed with SFT in 2014 and then underwent tumor removal, but relapsed just two months later. In July 2015, surgical resection alone was performed on the tumor in the abdominal wall, and the tumor recurred in the abdominal wall six months later. While surgery alone failed to improve the outcome, surgical removal combined with ^125^I seed implantation for the tumors in the left pelvic cavity and near the right iliac vessels showed a surprising benefit: the patient attained a complete remission, and no relapse was observed as of the end of follow-up, with a PFS of 43 months (**Table 1**). ^125^I seed implantation alone for the tumor behind the bladder continued to work: a complete remission was achieved, with a PFS of 35 months (**Table 1**). The PFS of 43 months and 35 months after seed implantation was far longer than the PFS of 2 months and 6 months after surgery alone. Seed implantation-yielded PFS in this case also showed notable superiority compared with the data of systemic drugs from previous research. Levard *et al.* described that the median PFS with doxorubicin alone and combined with ifosfamide was 4.0 months and 6.7 months, respectively (24). Park *et al.* evaluated the efficacy of temozolomide combined with bevacizumab in 14 patients with advanced SFT, and the median PFS was 10.8 months (25). A phase II clinical trial of sorafenib for treating five cases of advanced SFT showed a median PFS of 178 days (about 5.9 months) (26). Compared with surgery, seed implantation is more minimally invasive, and patients can recover more quickly; and it can be repeatedly performed if necessary. Patients have better tolerance to ^125^I seeds than to chemotherapy, which often causes severe side effects. Moreover, seed implantation costs less than chemotherapy and targeted drug therapy. However, seed implantation is a local therapy. If there is widespread metastasis, a combination of seed implantation and chemotherapy or targeted drug therapy may be a possible solution.

**Table 1:** The characteristics of treatment methods and efficacy.

Date	Tumor location	Treatment	Tumorresponse	PFS (months)
2015-7	the right wall of the abdomen	surgery	CR	6
	the left pelvic cavity	surgery+^125^I seed implantation	CR	43
	near the right iliac vessels	surgery+^125^I seed implantation	CR	43
2015-8/2015-9	behind the bladder	^125^I seed implantation	CR	35
2016-1	recurrent tumor in abdominal wall	^125^I seed implantation	CR	32
2018-10	recurrent tumor behind the bladder	^125^I seed implantation	CR	4
	recurrent tumor in abdominal wall	^125^I seed implantation	CR	4

In this report, the patient experienced repeated recurrence after surgery, but benefited from ^125^I seed implantation, which supports seed implantation as a potential solution to repeated local recurrence. As MSFT is very rare, there has been no more research than case reports, not to mention clinical randomized controlled trials. Our report could provide a reference for the treatment of MSFT.

## Data availability statement

The original contributions presented in the study are included in the article/supplementary material. Further inquiries can be directed to the corresponding author.

## Author contributions

ZG performed the bibliographic search and wrote the manuscript; ZG, HY, XD and JW revised the manuscript; YL, JZ and ZL took part to the equipment preparation and follow-up; HZ made the decision to submit the article for publication. All authors read and approved the final manuscript.

## Conflict of interest

The authors declare that the research was conducted in the absence of any commercial or financial relationships that could be construed as a potential conflict of interest.

## Publisher’s note

All claims expressed in this article are solely those of the authors and do not necessarily represent those of their affiliated organizations, or those of the publisher, the editors and the reviewers. Any product that may be evaluated in this article, or claim that may be made by its manufacturer, is not guaranteed or endorsed by the publisher.
